# Treating refractory corneal hydrops in a male patient with vernal keratoconjunctivitis and mental retardation: a case report

**DOI:** 10.1186/s12886-021-02241-6

**Published:** 2022-01-24

**Authors:** En-Jie Shih, Jung-Chia Lin, Kai-Ling Peng, Jiunn-Liang Chen

**Affiliations:** 1 School of Medicine, National Yang Ming Chiao Tung University, Hsinchu, Taiwan; 2 Department of Ophthalmology, Kaohsiung Veterans General Hospital, Kaohsiung City, 81362 Taiwan; 3 School of Medicine, Kaohsiung Medical University, Kaohsiung, Taiwan; 4 Chung Shan Medical University, Taichung, Taiwan; 5 Department of Pathology and Laboratory Medicine, Kaohsiung Veterans General Hospital, Kaohsiung, Taiwan

**Keywords:** Corneal hydrops, Keratoconus, Penetrating keratoplasty, Vernal keratoconjunctivitis, Intrastromal suturing, Tacrolimus, Case report

## Abstract

**Background:**

Keratoconus is the most common noninflammatory bilateral corneal ectasia. Vernal keratoconjunctivitis (VKC) and eye rubbing may be associated with keratoconus in children and young adults. Timely management of advanced keratoconus is important to improve visual quality. In addition, it is challenging to carry out VKC treatment with an intent to avoid the occurrence of punctate epithelial keratitis, ulceration, or corneal neovascularization on corneal grafts.

**Case presentation:**

We report the case of an 18-year-old male patient with a long-term history of mental retardation due to megalencephaly presenting with acute onset of corneal hydrops with prominent bulging and refractory steroid-induced glaucoma of the right eye. The topography of the right eye was unavailable due to advanced ectasia, and that of the left eye revealed central steepening with inferior-superior dioptric asymmetry. According to the clinical findings, the patient was diagnosed with keratoconus. Because of progressive corneal opacity and neovascularization, the patient underwent penetrating keratoplasty (PK) with combination of interrupted and intrastromal running suturing after receiving a preoperative subconjunctival injection of bevacizumab in his right eye, followed by lower eyelid correction. After surgery, the patient was treated with 0.1% tacrolimus dermatological ointment, 0.1% cyclosporine eye drops, artificial tears, and 0.5% loteprednol for keratoplasty and VKC. Repeated education on avoiding eye rubbing was offered to the patient. Two years after PK treatment, his best-corrected visual acuity of the right eye successfully improved from hand motion at 10 cm preoperatively to 6/20 postoperatively.

**Conclusions:**

Large-diameter PK with intrastromal suturing technique for advanced keratoconus could achieve better visual outcomes and avoid suture-related complications. In addition, tacrolimus dermatological ointment rather than tacrolimus topical eye drops or ointment showed satisfactory efficacy when combined with topical cyclosporine and steroid that no significant VKC reactivation were noted after PK.

## Background

Keratoconus is the most common noninflammatory bilateral corneal ectasia with an incidence of 1 per 2000 in the general population without sex-based differences [1]. The comorbidities, clinical presentation, and related non-corneal and corneal disorders of keratoconus have been described in the literature [1–4]. However, only few studies have reported the histological changes and electron microscopic findings observed in keratoconus [1, 5, 6].

Advanced corneal ectasia in keratoconus will deteriorate to sight-threatening complications such as corneal hydrops, corneal vascularization, or even corneal perforation, while management at these advanced stages becomes challenging without definitive guidelines. Various clinical treatment options have been reported for advanced corneal ectasia, including rigid gas permeable contact lens wearing, collagen cross-linking, and surgical approaches such as intrastromal gas injection, intrastromal corneal ring segment, keratoplasty, or wedge resection [1, 2, 4, 6, 7].

## Case presentation

An 18-year-old male patient visited our outpatient department with redness and pain in the right eye that lasted for 2 months. He was previously diagnosed with developmental delay, mental retardation, high myopia, and VKC accompanied by atopic dermatitis. He was presumptively treated for corneal ulcer under topical use of levofloxacin (Cravit®, Santen, Osaka, Japan) and 0.1% fluorometholone (FML®, Allergan, Dublin, Ireland) four times daily in a local clinic in his right eye. However, the patient developed quick progressive whitening and bulging of the cornea accompanied by symptoms of severe pain and photophobia. He was subsequently referred to our outpatient department for further evaluation.

At initial examination, the best-corrected visual acuity was hand motion at 10 cm in the right eye and 6/20 in the left eye. The intraocular pressure (IOP) was 35 mmHg in the right eye and 17 mmHg in the left eye upon Tono-Pen measurement. He was noted to have lagophthalmos with proliferative changes in the tarsal conjunctiva. Slit-lamp examination showed pictures of acute corneal hydrops and dense corneal opacity with clustered neovascularization around the limbus, even without views of the anterior chamber in the right eye. Visualization of the anterior chamber and iris was obscured due to corneal opacity.

**Fig. 1:**
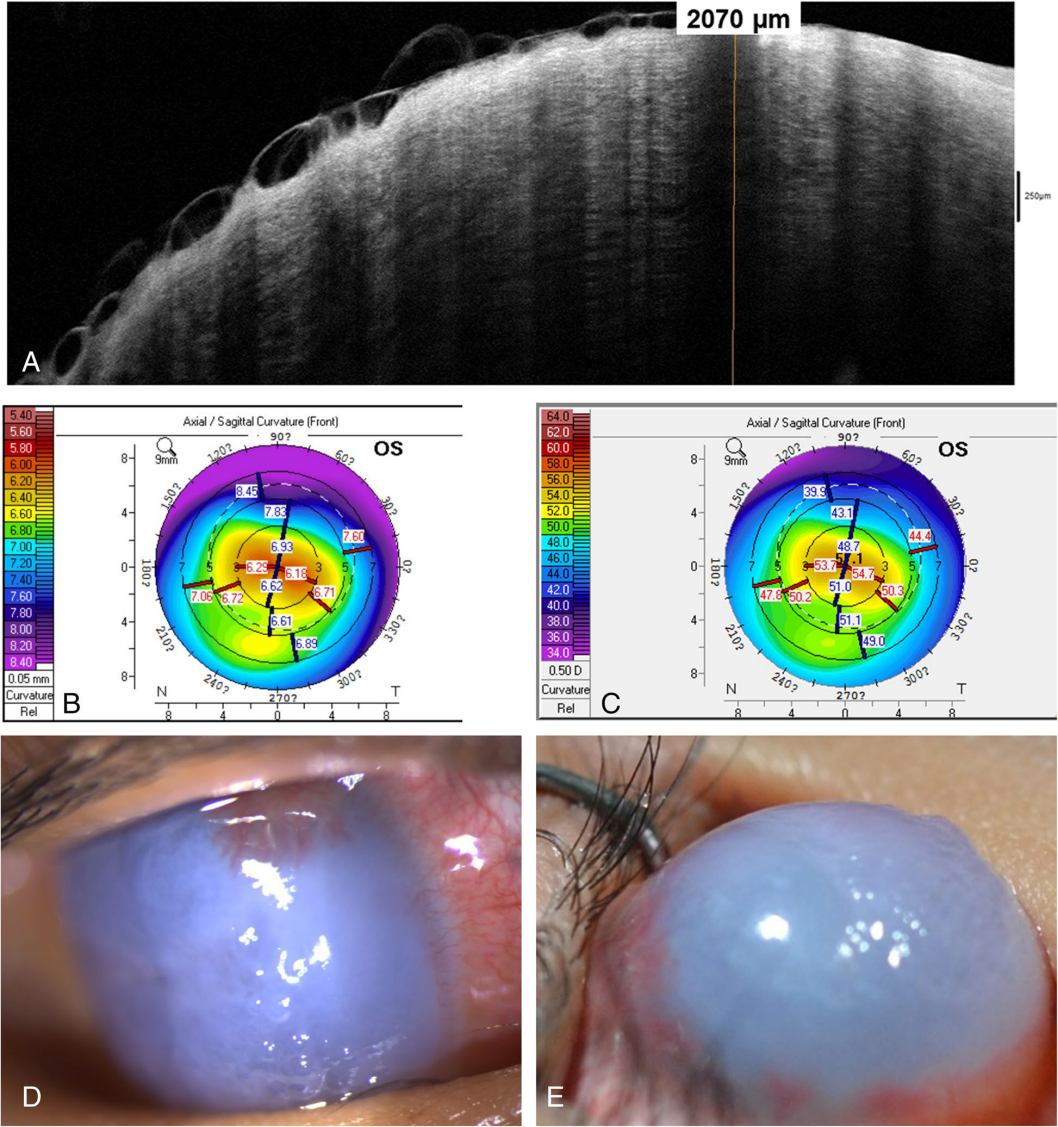
Pre-PK slit-lamp photography and ASOCT of the right eye and topography of the left eye. **A** ASOCT image of the right eye showing corneal hydrops with severe corneal edema. **B**, **C** Topographic images of the left eye showing central steepening with inferior-superior dioptric asymmetry. **D** Slit-lamp photographic image demonstrating severe corneal edema, total opacity with neovascularization of the right eye from limbus to limbus, epithelial bullae, and bulging eye with extensive corneal neovascularization superiorly. **E** Image taken 2 weeks later shows 360-degree peripheral corneal neovascularization. PK, penetrating keratoplasty; ASOCT, anterior segment optical coherence tomography

Anterior segment optical coherence tomography (OCT) findings of his right eye revealed a thickened cornea with superficial bullae and high refractivity of the entire layer (Fig. 1A). The central corneal thickness, axial length, and corneal curvature in the right eye/left eye were 2070 µm/587 µm, 29.76 mm/28.53 mm, and nonavailable/60 D, respectively. The topography of the right eye was not available due to severe ectasia, and the left eye presented typical features of keratoconus—central steepening and inferior-superior dioptric asymmetry (Fig. 1B and C). The IOP was 35 mmHg in the right eye. The IOP elevation in the right eye might have been related to marked corneal stromal edema with anterior chamber collapse or steroid-induced glaucoma. Culture tests for bacteria, viruses, and fungi were negative. The patient was finally diagnosed with advanced keratoconus with hydrops and secondary glaucoma.

**Fig. 2:**
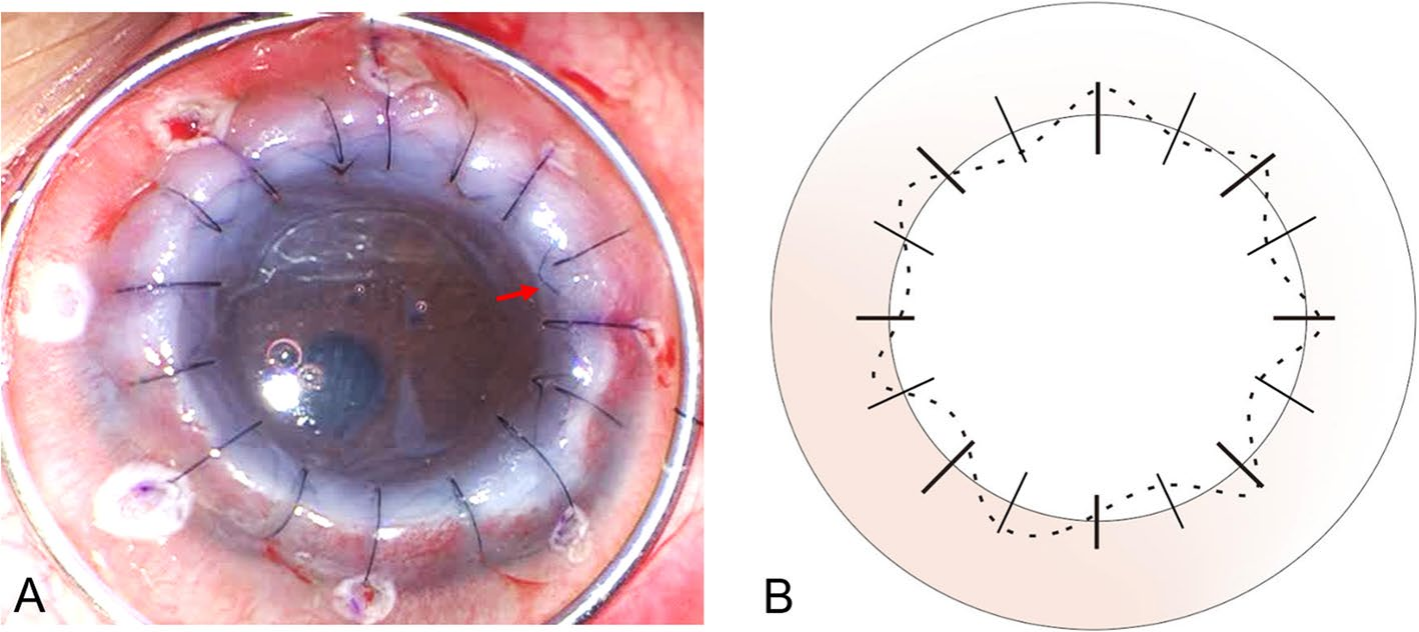
The surgical image and schematic diagram of the combined interrupted and continuous intrastromal suturing in PK. **A** Large-diameter PK with a combination of interrupted and intrastromal running suturing (red arrow). **B** The technique of combined interrupted and continuous intrastromal suturing was performed first with eight radial interrupted sutures (coarse solid lines), followed by intrastromal sutures (dotted line). The needle’s entry and exit points were the same to ensure that the intrastromal sutures passes over the intrastromal and deep layers rather than the epithelial layer alone. Finally, the graft-host junction was strengthened by additional eight sutures (fine solid lines). PK, penetrating keratoplasty

Because of mental retardation and lagophthalmos, the patient was unable to cooperate in wearing a rigid contact lens. In addition, it was challenging to inject intracameral gas due to limbus-to-limbus total opacity of the cornea (the details of the iris and pupil in the anterior chamber were totally obscured). As for corneal ectasia, the corneal curvature was too severe to receive collagen cross-linking. Three days after the initial evaluation at our hospital, there was rapid progression of corneal ectasia with total opacification (Fig. 1D). Two weeks later, 360-degree peripheral corneal neovascularization was noted (Fig. 1E) with poor IOP control. The patient then received preoperative subconjunctival injection of bevacizumab (Avastin®, Genentech Inc., South San Francisco, CA, US) for severe peripheral corneal neovascularization and therapeutic PK with a donor diameter of 9.5 mm with combination of interrupted and unique intrastromal running suturing technique (Fig. 2A and B). The technique was performed first with eight radial interrupted sutures, followed by intrastromal sutures. The needle’s entry and exit points were the same to ensure that the intrastromal sutures passed over the intrastromal and deeper layers rather than the epithelial layer. Finally, the graft-host junction was strengthened using eight additional interrupted sutures (Fig. 2A and B).

**Fig. 3:**
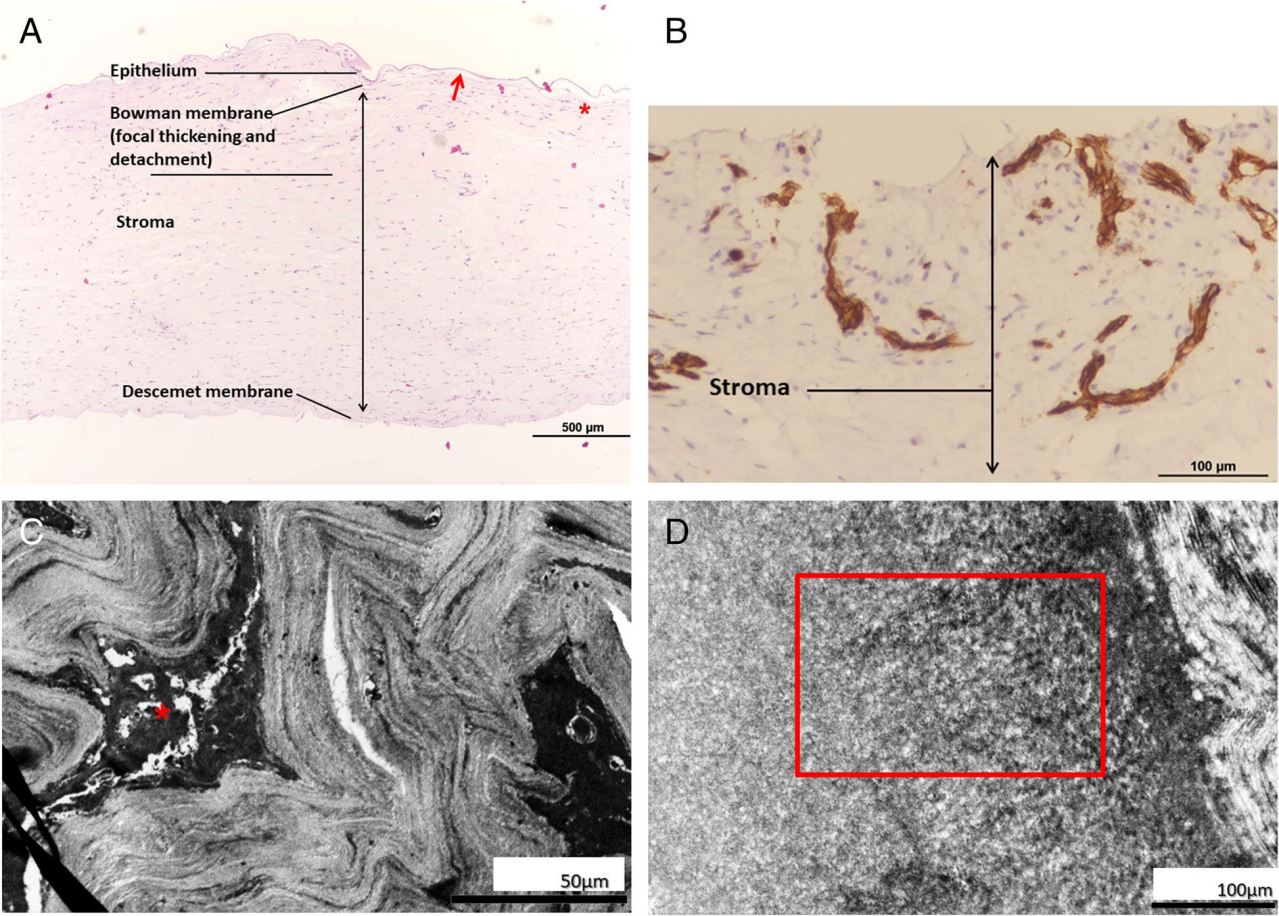
Pathologic findings obtained using light microscopy and transmission electron microscopy. **A** H&E staining image at 40× magnification showing Bowman’s layer with focal thickening (red star) and detachment (red arrow). **B** CD31 staining image at 200× magnification showing focal vascularization of the stroma (red star). **C** Transmission electron microscopy image of the stroma showing focal vascularization (red star) (original magnifications: 1400×). **D** Transmission electron microscopy image showing some equivocal collagen fibril disarray in the Bowman’s layer (red box) (original magnifications: 1400×). H&E, Hematoxylin and eosin. *We used light microscopy Eclipse E600 (Nikon Instruments Inc., Tokyo, Japan) with Nikon DS-Ri2 camera, and software of NIS-Elements documentation. The transmission electron microscopy JEM-1400 (JEOL Inc., Tokyo, Japan) is equipped with a CMOS camera, Silicon drift detectors, filters of NCB11, ND8, ND32, and software of RUBY 2 CCD

Histopathological examination of the recipient cornea under light microscopy showed focal thickening with detachment of the Bowman’s layer and focal vascularization of the stroma (Fig. 3A and B). Collagen fibril disarray over Bowman’s layer and focal vascularization of the stroma were observed by transmission electron microscopy (Fig. 3C and D).

**Fig. 4:**
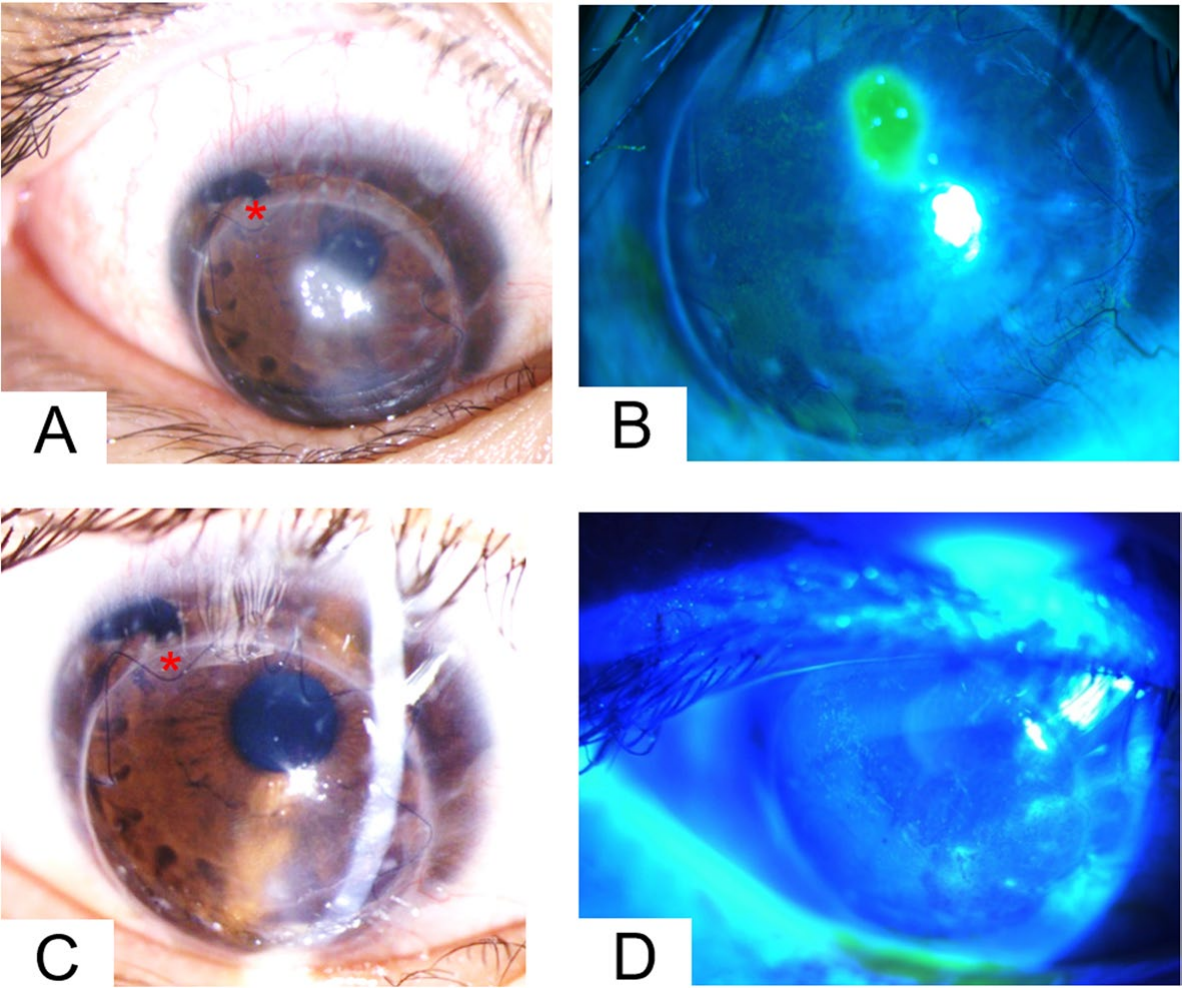
Postoperative external photography of the right eye after penetrating keratoplasty. **A**, **B** Images taken at 3 months after PK showing a persistent central corneal epithelial defect with vernal keratoconjunctivitis of the right eye. **C**, **D** Images taken 6 months after PK and 3 months after epiblepharon correction showing no corneal opacity or edema. The image also shows resolved trichiasis and epithelial defect with minimal superficial punctate keratitis. No suture-related complications, such as suture infiltration and loosening were noted over our intrastromal continuous running sutures (red star). PK, penetrating keratoplasty

Postoperatively, the patient presented with a persistent paracentral shield ulcer over the corneal graft (Fig. 4A and B). The epithelial defect persisted for weeks due to refractory VKC and mild trichiasis despite aggressive lubrication. After blepharoplasty and application of 0.1% tacrolimus dermatological ointment (Protopic®, Astellas Pharma, Tokyo, Japan) on the eyelid, the shield-shaped epithelial defect healed thereafter. The postoperative IOP in the right eye was within the normal range. Eye drops of 0.1% cyclosporine (Ikervis®, Santen, Osaka, Japan) QID, 0.5% loteprednol (Lotemax®, Bausch & Lomb, Quebec, Canada) QID, levofloxacin (Cravit®) QID, and artificial tears (Artelac®, Bausch & Lomb, Quebec, Canada; Duratears®, Alcon Laboratories, Inc., Fort Worth, Texas, USA) with 0.1% tacrolimus ointment (Protopic®) BID over the eyelid were offered initially and tapered gradually within 18 months after surgery. The patient and his family were continuously educated on how to avoid eye rubbing. Two years after corneal transplantation, the patient’s best-corrected visual acuity improved from hand motion at 10 cm to 6/20 in the right eye, and the graft remained clear with no apparent epithelial defect (endothelial cell count, 1416; central corneal thickness, 556 μm) (Fig. 4C and D).

## Discussion and conclusions

Previous studies have revealed that keratoconus coexists with comorbidities such as atopy, Down syndrome, Ehlers-Danlos syndrome, Marfan’s syndrome, pseudoxanthoma elasticum, and mitral valve prolapse [1, 3, 7]. Other reports on combined ocular disorders include congenital cataracts, blue sclera, retinitis pigmentosa, and VKC [1, 3, 7]. Ectatic disorders, such as keratoconus, pellucid marginal degeneration (PMD), and keratoglobus, share overlapping ocular features with corneal protrusion, localized corneal thinning, and severe uncorrectable refractive problems. Some case reports have indicated that the natural courses of keratoconus and PMD may result in the development of keratoglobus according to a series of topographies [8, 9]. The most common type of corneal ectasia is keratoconus, which usually occurs during puberty and presents with progressively central or paracentral corneal thinning with cone-shaped protrusion. Environmental risk factors include eye rubbing and contact lens use. In our case, corneal neovascularization with acute onset of hydrops was noted as the late and progressive stage of corneal ectasia [8]. Due to VKC and trichiasis since birth, our patient, who had a history of mental retardation, kept rubbing his eyes uncontrollably and had a delay in his treatment. His corneal ectasia was compatible with extremely high myopia, irregular astigmatism, and poor vision.

Histological staining of hematoxylin-eosin in keratoconus has shown characteristics of breaks in Bowman’s and Descemet’s membranes [10]. Focal disruption of Bowman’s layer, scarring with central or peripheral vascularization of the stroma, and unremarkable Descemet’s membrane have been reported [1]. Transmission electron microscopy of corneal ectasia is uncommon. In our case, we further assessed the corneal sample under transmission electron microscopy for equivocal collagen fibril disarray in Bowman’s layer and focal vascularization of the stroma, consistent with the findings reported in previous studies [5].

Treatment for mild to moderate keratoconus usually begins with the use of eyeglasses, contact lens correction, or intracameral air injection. In advanced cases, surgical intervention through keratoplasty is necessary [1, 11]. Current keratoplasty procedures include PK, deep anterior lamellar keratoplasty, or lamellar keratoplasty with some degree of modification based on epikeratoplasty [1, 12, 13]. Ideal transplants for keratoconus ranged from 8.0—8.5 mm [14] to avoid larger graft-related complications of persistent epithelial defect, graft rejection, or secondary glaucoma [15]. However, the indications of large-diameter PK (8.75—10.0 mm) have been reported for cases with peripheral corneal pathology, such as keratoconus or PMD, to replace excised peripheral pathologic cornea and produce less astigmatism to achieve better visual outcomes [15]. In our keratoconus case, we chose a large-diameter donor cornea (9.5 mm) to cover the excised periphery and rescue the patient’s vision.

Previous studies have reported various severities of suture-related complications and graft rejection-like reactions, including suture infiltration and loosening in keratoconus eyes, after keratoplasty [16, 17]. Keratoconus eyes with post-PK episodes of VKC reactivation further increased the rate and severity of suture-related problems, such as suture abscess and suture-tract vascularization [16]. To avoid the aforementioned complications and problems that occurred in our case, we combined interrupted sutures with intrastromal suturing to bury the sutures in the corneal stromal layer without exposure. The interrupted sutures were removed 2 weeks after keratoplasty to prevent suture hypersensitivity due to the exposed suture materials. Even if all interrupted stitches are removed during the early postoperative period, intrastromal suturing is sufficient to maintain tectonic strength. A similar technique has been introduced in cataract surgery to reduce the risk of wound infection and radial keratotomy to maintain stable refraction [18, 19].

Topical management of VKC includes histamine receptor blockers, mast cell stabilizers, steroids, and calcineurin inhibitors (cyclosporine or tacrolimus) [20]. Tacrolimus eye drops and ointment have been reported as promising treatments for severe VKC in some European and Asian countries [21, 22]. However, they are only available in certain countries; thus, topical tacrolimus dermatological ointment is another option for VKC. A recent study in Taiwan showed good efficacy of tacrolimus dermatological ointment in ten patients diagnosed with refractory VKC under two-year follow-up, in which reduced size of the papilla, improved hyperemia, and shield ulcer healing with reepithelization were noted after 0.1% tacrolimus dermatological ointment [23]. In addition, 0.05–0.1% cyclosporine eye drops have also been reported to relieve symptoms and signs such as tearing, discharge, size of the papilla, conjunctival hyperemia, and corneal neovascularization in VKC [24, 25]. These topical agents are not only safe and effective for the treatment of VKC, but also reduce the dosage of steroids to avoid long-term side effects such as secondary glaucoma. In our case, after applying tacrolimus dermatological ointment combined with eye drops of cyclosporine, steroid and artificial tears postoperatively, the giant papilla became flatter without shave excision, and the shield ulcer did not recur.

Our case indicates that large-diameter PK with intrastromal running suturing technique for advanced keratoconus could achieve better visual outcomes and avoid suture-related complications. In addition, tacrolimus dermatological ointment rather than tacrolimus eye drops or ointment showed satisfactory efficacy when combined with topical cyclosporine and steroid that no significant VKC reactivation were noted after PK in our case.

## Data Availability

All data generated or analyzed during this study are included in this published article.

## Abbreviations

PMD: pellucid marginal degeneration
PK: penetrating keratoplasty
ASOCT: anterior segment optical coherence tomography
